# Integrated Primary Care and Public Health: A Case Study of Multidisciplinary Rehabilitation and Lifestyle Intervention in Persons With Post-COVID-19 Condition

**DOI:** 10.3389/ijph.2022.1605287

**Published:** 2022-10-28

**Authors:** Teja Oblak, Nataša Vidnar, Rade Pribaković Brinovec

**Affiliations:** ^1^ Epidemiology and Cancer Registry, Institute of Oncology Ljubljana, Ljubljana, Slovenia; ^2^ Community Health Centre Adolf Drolc Maribor, Maribor, Slovenia; ^3^ Centre for Promotion and Prevention Programme Management, National Institute of Public Health, Ljubljana, Slovenia

**Keywords:** primary health care, public health, post-COVID-19 condition, rehabilitation, lifestyle change

After 40 years of Alma-Ata declaration and shortly after Astana declaration, primary health care (PHC) faced a big test with the COVID-19 pandemic [1]. Nevertheless, the system rapidly adjusted and transformed in line with the pandemic response and struggled to provide essential as well as COVID-19-related services [1]. Despite taking over a double burden, lots of criticism was raised against lower accessibility of PHC for people with non-communicable diseases (NCD) and vulnerable groups, while the pandemic was soon revealed to be rather a COVID-19 syndemic [2].

In this article, we discuss experience of PHC during and after COVID-19 in Slovenia, a small country in Central Europe. The Slovenian rapid and effective dual-track approach of PHC to the pandemic was possible due to the integrated primary care model [3], which has been intertwined with public health, health promotion and prevention for the last 20 years [3, 4]. After first declines in COVID-19 cases, this intertwinement enabled PHC also to detect the most burning needs of local communities and develop new services [5].

In Slovenia, PHC is delivered by the Community-based Primary Healthcare Centres (CHC) located throughout the country and by individual or group practices of private practitioners. The basic model is an integrated care model, providing prevention and care coordination for patients with chronic diseases by the team of family physician, assistant nurse and registered nurse [3]. From 2002 on, the multidisciplinary Health Promotion Centres (HPC) were incorporated into CHC [4]. Their role was to carry out cross-sectoral adult prevention and health promotion for NCD, especially targeting vulnerable groups and hard-to-reach people in local communities [4].

During the COVID-19 pandemic in Slovenia, majority of prevention and health promotion activities as well as other non-essential services at PHC were discontinued [5]. Personell at the HPC was transfered to other workplaces inside PHC or hospitals. Nevertheless, apart from the newly-set up ambulatory clinics to treat and follow-up COVID-19 patients and provision of other non-COVID-19 services, PHC in Slovenia carried out the majority of the most important public health measures on a population level: infection containment at PHC and social care institutions, mass testing, mass vaccination, personell education, patient education, health promotion and prevention [5].

Becoming a COVID-19 syndemic, the SARS-CoV2 infection has interacted with an array of NCD and clustered among the socio-economically deprived people and other vulnerable groups [2]. Due to closures of non-essential services and prevention activities causing a fall in number of referrals, diagnostics and treatments for NCD, the onset of new NCD and their exacerbations could not have been detected in their earlier phases [6]. According to the Slovenian periodic panel web survey on the pandemic fatigue named SI-PANDA, nearly 35% of people with non-COVID-19 symptoms didn’t consult a doctor in order to avoid contracting SARS-CoV2 or not to burden healthcare system in 2020–21 [7]. Due to pandemic fatigue, people’s enthusiasm about healthy lifestyle whithered away, while nearly 39% of respondents reported less physical activity, 28% ate more unhealthy foods, and 18% more of them smoked and drank alcohol than before the pandemic, especially young adults [7]. Among respondents with NCD, almost one third reported gaining weight [7]. Apart from that, the survey showed that nearly half of COVID-19 convalescents reported various symptoms persisting more than 3 months after infection, which significantly limited their performance at work, home and influenced relationships among two thirds of them [7]. Post-COVID-19 condition (or long Covid) is a cluster of respiratory, musculoskeletal and neuropsychiatric symptoms, occuring usually 3 months from the onset of COVID-19 symptoms that last for at least 2 months and cannot be explained otherwise, and have an impact on everyday functioning [8]. The unmet health needs of many people with the post-COVID-19 condition were also observed in ambulatory care at CHC Maribor, while tertiary rehabilitation capacities throughout Slovenia have been limited to treating only patients with severe COVID-19 discharged from the intensive care units.

By a bottom-up approach, multidisciplinary team from the HPC at CHC Maribor in collaboration with primary care pulmologists and family doctors self-designed the first post-COVID-19 rehabilitation programme in the country, stemming from their pre-pandemic work in health promotion and prevention. In June 2021, the comprehensive pilot programme started in a heterogenous group of people with or without previous NCD who reported debilitating post-COVID-19 symptoms and various level of functional disability, which persisted more than 3 months after infection, despite being treated at home, at the hospital or intensive care unit. The goal-oriented, individually tailored, free-of-charge programme consisted of 15-week long workshops twice per week including physiotherapy, psychoeducation, nutritional support and counselling to stop risky health behaviours. For the multidisciplinary team of health promotion proffesionals, the main goal was to help participants regain the same functional ability as before the COVID-19 syndemic and reach a long-standing lifestyle change. After 1 year of the ongoing pilot rehabilitation programme, all hundred participants with post-COVID-19 condition gained important functional progress measured by objective periodic assessments, higher independency in everyday activities and healthier lifestyle, while they self-reported higher resilience to stress, better mental health and higher ability to concentrate. In addition, their advancement had a positive influence on their families.

However, comprehensive evaluation and quality assessment of this pilot programme showed some space for improvement, namely in structuring the groups by similar ages and functional needs, assessing the psychological and quality-of-life outcomes by validated questionnaires and directing participants with severe COVID-19 complications or deteriorations in NCD into other subspecialised rehabilitation beforehand. After concluding the programme, participants stayed motivated to maintain a healthy lifestyle and participate at other health preventive activites at the CHC, though they mostly displayed a need for longterm mental health follow-up.

Devising a comprehensive, multidisciplinary post-COVID-19 rehabilitation programme encompassing individually tailored primary-level rehabilitation and public health intervention with promising outcomes has only been possible in the integrated primary care deeply intertwinned with public health and oriented toward local communities and vulnerable groups [3, 4]. Such multidisciplinary approach is significantly strenghtening the resilience of individuals, local communities and population as a whole for the future syndemics [9]. This is especially important for regions with prevalent health inequalities, such as the Maribor Municipiality and wider Eastern Slovenian region. Recently, a national-wide programme of the post-COVID-19 rehabilitation at PHC with the longterm systemic funding and rigorous quality control has been set up by the National Institute of Public Health of Slovenia.

This successful case study is in conjuction with the international recommendations of World Health Organisation and other experts how to tackle the COVID-19 syndemic efficiently [9, 10]. Primary care health promotion and prevention programmes should become essential services [5] as well as rehabilitation and stay operating during future syndemics, while proffesionals from its multidisciplinary teams and local communities should be included in decision-making process about the national syndemic response.
